# Factors associated with pharmacological treatment in children with attention-deficit/hyperactivity disorders: a retrospective study of a series of 77 cases in a single third-level reference Centre in Apulia region

**DOI:** 10.1186/s13052-023-01560-2

**Published:** 2023-11-14

**Authors:** Simone Amendola, Isabella Fanizza, Sara Scoditti, Marta De Rinaldis, Antonio Trabacca

**Affiliations:** Scientific Institute I.R.C.C.S. “E. Medea”- Unit for Severe Disabilities in Developmental Age and Young Adults (Developmental Neurology and Neurorehabilitation) - Piazza “A. Di Summa”, Brindisi, 72100 BR Italy

**Keywords:** Psychopharmacology, Medication, Drug prescription, Adherence, Treatment persistence, Discontinuation

## Abstract

**Background:**

The present study analysed data on children and adolescents with a diagnosis of attention-deficit/hyperactivity disorder (ADHD) who were referred to the ADHD reference centre of Scientific Institute IRCCS *E. Medea* (Brindisi, Italy) for ADHD pharmacotherapy initiation and monitoring overtime. The main aim of the study was to examine differences in pharmacological treatment status (i.e., treatment continuation vs discontinuation) between patients.

**Methods:**

Seventy-seven children and adolescents (mean age at pharmacotherapy initiation = 9.5, standard deviation = 2.6) with ADHD received drugs treatment for ADHD at the reference center between January, 2013 and May, 2022. Demographic and clinical data were obtained from the Italian Registry for ADHD and medical records. Child Behavior Checklist (CBCL) available data were used.

**Results:**

Pharmacological treatment status was examined for patients (*n* = 63) with at least 12 months of follow-up after the first pharmacological treatment for ADHD. After starting pharmacotherapy treatment, 77.8% (*n* = 49) patients were still on treatment whereas 22.2% (*n* = 14) discontinued it. No between group difference were observed in demographic and clinical data except for the intelligence quotient/intellectual disability and rule-breaking behavior (*n* = 40).

**Conclusions:**

This study stressed the need of periodical assessments, monitoring difficulties with treatment and/or reasons for poor treatment compliance to provide individualized care.

## Background

Attention-Deficit/Hyperactivity Disorder (ADHD) is a neurodevelopmental disorder characterized by age-inappropriate levels of attention and/or impulsivity lasting for at least 6 months [1]. In order to diagnose ADHD, symptoms must first appear in childhood and affect daily functioning and different contexts (e.g., family and school) and cannot be better explained by another disorder [2]. Willcutt et al. [3] reviewed findings from 86 studies for a total of 163,688 children and adolescents and found an ADHD prevalence of 5.9% in children and 7.1% in adolescents. ADHD is generally associated with poor academic outcomes, peer relationship problems, and family stress and difficulties [4–9]. Its clinical presentation can be differentiated in a predominantly inattentive subtype, a predominantly hyperactive/impulsive subtype, and a combined subtype [1]. Further, ADHD frequently co-occurs with internalizing and externalizing disorders. For example, girls with ADHD are significantly more likely to show comorbid internalizing (anxiety, depression) and externalizing (oppositional defiant disorder, conduct disorder) psychopathology compared with girls without ADHD [10]. Nearly seven out of 10 young patients with ADHD met the diagnostic criteria for at least one comorbid psychiatric disorder, with learning, sleep and oppositional disorders being the most frequent ones [11]. Previous studies also explored psychopathological subtypes in children and adolescents with ADHD demonstrating the existence of different groups, i.e., low symptoms, mainly externalizing, mainly internalizing, and high symptoms of psychopathology [12, 13].

### ADHD characteristics and its treatment in Italy

ADHD in the Italian young population shows a prevalence of 2.9% (range: 1.1–16.7%). Prevalence of clinically confirmed ADHD diagnoses drops to to 1.4% (range: 1.1–3.1%) [14].

SINPIA (Italian Society of Childhood and Adolescent Neuropsychiatry) [15] Guidelines recognize a multimodal approach to ADHD as the most suitable intervention. It consists of non-pharmacological interventions directly involving patient, family and school (e.g., cognitive-behavioral therapies, parent and teacher training), while pharmacotherapy should be considered in the most severe cases of ADHD, when patients do not respond to psycho-behavioral treatments [15, 16].

The Italian National Registry for ADHD was set up in 2007 to monitor ADHD drugs use in childhood and adolescence and assess safety, benefits and compliance [17–19]. Changes in pharmacological treatment of ADHD in Italy over time has been previously discussed [20] and data from this registry were analysed in relevant publications [21–23]. ADHD prevalence was mainly stable (1.2% in 2007 and 1.1% in 2010) whereas incidence of new cases decreased in the 2007–2010 period (0.06% in 2008 and 0.03% in 2010) [21]. One third of patients diagnosed with ADHD did not receiv any treatment (watchful waiting approach) while the remaining two thirds received some form of treatment (around 73% yearly). Prevalence of multimodal treatment increased overtime from 7% (in 2007) to 16.7% (in 2010) whereas psycho-behavioral interventions decreased from 65 to 58.2%. Among the latter, parent training was prescribed in 66.1% of cases whereas cognitive-behavioral therapy in 24.8%. Finally, children aged 6–10 years made up almost 60% of total ADHD cases. Similar findings were observed with samples of patients evaluated by 18 ADHD regional reference centres in Lombardy (Italy) [23, 24].

Patients on drugs more commonly presented symptoms of clinical severity (as evaluated by clinicians using the item of the Clinical Global Impression), combined type of ADHD, clinical dis-attention and hyperactivity (as reported by parents) compared to subjects receiving psychological interventions only [23]. In a recent update [25], comorbidity (i.e., oppositional defiant disorder, intellectual disability, tic and coordination disorder) was more likely found in patients on pharmacotherapy than psychological interventions.

### Treatment (dis-)continuation and psychopathology

Identifying factors associated with treatment (dis-)continuation may have important clinical implications for individually tailored interventions [26].

Adherence to, and persistence of, ADHD pharmacological treatment are generally low among young people with ADHD and vary between 40 and 70% and 30–50%, respectively [27–29]. Low adherence and persistence is associated with several factors, including older age, being male, late diagnosis of ADHD, family history for ADHD, high-level of paternal education, parental separation, absence of comorbidities, multiple daily doses, high doses, side effects, lack of efficacy, stigma, higher ADHD symptoms [2, 26, 30–38].

Few studies examined the relationship between psychopathological symptoms and (dis-)continuation of drug treatment for ADHD reporting mixed findings. Atzori and colleagues [26] retrospectively compared 134 patients attending the Centre for Pharmacological Therapies in Children and Adolescents Psychiatry (Cagliari, Italy) between 1998 and 2005, and taking methylphenidate with a follow-up of at least 3 years. At the 3-years follow up, not only the significant effects of younger age, being female and not living with both parents, but also comorbidities were predictive factors of therapy continuation. On the other hand, lack of comorbidities (besides the significant effect of older age) was associated with therapy discontinuation due to functional remission. Finally, discontinuation for other reasons (e.g., poor compliance, reduced efficacy) was not associated with comorbidity but it was associated with older age at therapy start and an hyperactive subtype of ADHD [26]. The relationship between psychiatric comorbidity and persistence of ADHD therapy has been confirmed by a retrospective study using Korean Health Insurance data from 2007 to 2011 [39].

Other studies which did not differentiate between discontinuation reasons (i.e., remission versus other reasons) showed no association between therapy continuation and psychopathology. For example, Palli et al. [35] did not find any evidence of an association between ADHD therapy continuation and comorbid psychopathology using Medicaid Analytic eXtract data from Texas, New York, California and Illinois (United States) between 2003 to 2005. By contrast, continuation of ADHD therapy was associated with prescriptions of other psychotropic drug classes (other than stimulants). Willingness to take medications could explain the association between continuation of ADHD pharmacotherapy and prescriptions of other psychotropic drugs, despite findings are mixed. In 118 children with ADHD who were referred to a child psychiatry clinic in Iran in 2018, Safavi et al. [40] showed that comorbidity was not associated with adherence to ADHD pharmacotherapy while ADHD children with a family history of psychotropic drug use showed a higher adherence to ADHD pharmacotherapy compared to those with no family history. Similarly, another study [41] on 50 children attending a tertiary care centre in Mumbai (India) did not find any evidence of associations between adherence to ADHD pharmacotherapy and conduct disorder, oppositional defiant disorder and emotional problems, but adherence was negatively correlated with ADHD severity. A recent retrospective study [42] of adherence to methylphenidate in Japan demonstrated no association between adherence and concomitant use of antipsychotics, antidepressants and anxiolytics. Finally, Cheung et al. [43] reported no significant univariate association between methylphenidate treatment adherence at 2 years and the five Diagnostic and Statistical Manual of Mental Disorders - oriented scales of the Child Behavior Checklist (CBCL), and symptoms of ADHD, in a sample of 264 children in the Netherlands.

### Study aims and hypotheses

The first aim of this study was purely descriptive, namely reporting on demographic and clinical characteristics of children and adolescents aged 5 to 17 years who received drug treatment for ADHD at the reference center Scientific Institute IRCCS “*E. Medea*” (Brindisi, Italy) between January, 2013 and May, 2022.

The second aim of this study was to contribute to scientific knowledge on the association between (dis-)continuation of ADHD pharmacotherapy, demographic and clinical characteristics, in particular symptoms of psychopathology when initiating ADHD pharmacotherapy. Considering previous mixed findings on the relationship between treatment (dis-)continuation and psychopathology, the study was exploratory in nature and we expected either a higher incidence of psychopathology in patients who continued pharmacotherapy compared to those who did not it [26, 39] or no between-group differences [35, 40–43].

## Methods

### Participants and procedure

This retrospective study included children and adolescents aged 6–17 who were referred to Scientific Institute IRCCS “*E. Medea*” in Brindisi (Italy) – one of the Apulia reference prescription centres for ADHD pharmacological treatment – between January 2013 and May 2022. Prescription centres are responsible for confirming ADHD diagnosis and monitoring the pharmacological therapy over time. In this Italian region, eight reference prescription centres were accredited as specialist centres for diagnosis and treatment of ADHD [44]. Patients were included in this study if they were enrolled in the Italian National Registry for ADHD and received a prescription for methylphenidate (MPH) from the reference center Scientific Institute IRCCS “*E. Medea*” in Brindisi (Italy). No other inclusion/exclusion criteria were used.

Written informed consent for drug administration and analysis of clinical data for scientific research was obtained from both parents and patients. Patients are hospitalized to receive their first drug administration, the so-called “testing dose” (5 mg of immediate-release methylphenidate) to monitor for potential side effects and vital signs (e.g., brain and cardiac activity). If there are no relevant side effects, patients start long-term treatment with modified-release methylphenidate, and a first follow-up takes place after a month. Modified-release methylphenidate is generally administered at a dose ranging from 0.3 to 0.5 mg/kg until a maximum of a single dose of 60 mg/die is reached. In some cases, 5 mg of immediate-release methylphenidate is also administered in the afternoon to help patients do their homework. The drug dose is determined based on the improvement seen in child behaviour and psychosocial functioning. Most patient have an annual “drug holiday” (of approximately two/three months) during summer according to their symptomatology, in order to decrease the effects of medication on height and weight and assess the need for continuing medication during the early month(s) of the new school year. The research was approved by the local Ethics Committee, “Research Ethics Committee – IRCCS Istituto Tumori Giovanni Paolo II - Bari (Italy).”

### Measures

The following demographic and clinical data was obtained from the Registry for ADHD and medical records: age at treatment initiation, sex, hometown, months between diagnosis and pharmacotherapy, months of pharmacotherapy, intelligence quotient (IQ), subtype of ADHD diagnosis, comorbidity and pharmacological treatment status. IQ was measured using the Wechsler Intelligence Scale for Children (WISC-III and -IV), the Leiter International Performance Scale – Revised (Leiter-R), and the Wechsler Preschool and Primay Scale of Intelligence (WPPSI-III). ADHD diagnosis subtype and comorbidities were diagnosed according to the DSM-IV-TR and DSM-5.

Pharmacological treatment status was defined as “on-treatment” and “treatment discontinuation” as of May 2022, and follow-up data of at least 12 months after the first administration of ADHD drug therapy had to be available.

The Child Behavior Checklist (CBCL/6–18) [45, 46] was used to explore emotional and behavioural symptoms, competencies and adaptive functioning as reported by parents. Items are scored on a three-point scale (0 = not true, 1 = somewhat or sometimes true, and 2 = very or often true) and refer to symptoms presentation in the preceding 6 months. Internalizing and externalizing broad band symptoms dimensions were empirically developed from the CBCL (Achenbach & Rescorla, 2000). All CBCL scales have a mean T-score of 50 and standard deviation of 10 and different norms are provided for each gender across different age ranges. Previous research showed the usefulness of syndrome scales for a complete and accurate assessment of young patients [47–49]. In this study, we examined the most recent CBCL completed before the first methylphenidate administration.

### Statistical analysis

Descriptive statistics (number, frequency, mean and standard deviation) were computed to explore the characteristics of the sample and subgroups according to pharmacological treatment status. Subgroup analysis based on pharmacological treatment status included participants (*n* = 63) with at least 12 months of follow-up data after the first administration of ADHD drug therapy as of May 2022.

Between-group differences according to pharmacological treatment status were analysed using the Chi-square test and Student’s t-statistics for group comparison on categorical and continuous variables, using Cramer’s V and Cohen’s d as estimate of effect size, respectively. When the assumption on equality of variance (tested using Levene’s test) was violated (*p* < 0.05 or approaching significance at *p* < 0.10) Welch homogeneity correction was applied. Effect size for Cramer’s V was interpreted as moderate if between .2 and .6, and strong if higher than 0.6. For Cohen’s d, it was interpreted as moderate if between .5 and .8, and strong if higher than .8.

Subsequently, two multivariate logistic regression models were used to test associations between pharmacological treatment status and variables of interest (*model 1*) and psychopathology as measured by the CBCL subscales (*model 2*), including variables whose bivariate associations were significant (*p* < 0.05) or approaching significance (*p* < 0.10) based on subgroup analysis. Model 2 included only participants (*n* = 40) for whom CBCL data were available. For some children and adolescents, CBCL data were missing as they had been evaluated for ADHD diagnosis by child and adolescent neuropsychiatrists and were later referred to Scientific Institute IRCCS “*E. Medea*” for their first prescription of ADHD pharmacological therapy and monitoring over time. In this cases, no new (complete) diagnostic assessment is carried out. During a preliminary analysis, differences between participants, whose data was considered for subgroup comparisons (*n* = 40 with and *n* = 23 and without CBCL evaluation) were explored, and no differences in demographic and clinical characteristics were found.

All analysis were performed using JASP version 0.16.0.0 [50] and significance was set at *p* value < 0.05.

## Results

ADHD was diagnosed at a mean age of 8.8 years (Table 1). The time between diagnosis and pharmacotherapy initiation was generally 8 months. 26% of patients lived in the same town where the Scientific Institute was (i.e., Brindisi), and the rest in other Apulia provinces (i.e., 37.7% in Lecce, 20.8% in Taranto, 11.7% in Bari, 3.9% in other provinces).

**Table 1:** Demographic and clinical characteristics of total sample and pharmacological treatment status subgroups

	Total sample (*N* = 77) M (SD)	In treatment (*n* = 49) M (SD)	Treatment discontinuation (*n* = 14) M (SD)	*t / χ*^*2*^ (df)	Cohen’s *d* / Cramer’s *V*
Age,	9.5 (2.6)	9.3 (2.6)	10.2 (2.8)	−1.213 (61)	.37
Males, *n (%)*	66 (85.7)	43 (87.8)	12 (85.7)	0.041 (1)	.03
Age at diagnosis	8.8 (2.6)	8.6 (2.4)	9.2 (2.9)	−0.775 (61)	.24
Months between diagnosis and pharmacotherapy ^b^	8.4 (11.8)	7.4 (11.2)	11.9 (15.2)	−1.046 (17.4)	.34
Months of pharmacotherapy ^b^	36.3 (28.5)	50.8 (25.3)	12.2 (14.2)	7.361 (38.61) *	1.88
ASL of residence in the same city as Scientific Institute location, *n (%)*	20 (26)	13 (26.5)	3 (21.4)	0.150 (1)	.05
*Diagnosis, n (%)*				1.552 (1)	.16
ADHD-C	71 (92.2)	44 (89.8)	14 (100)		
ADHD-H	1 (1.3)	–	–		
ADHD-I	5 (6.5)	5 (10.2)	0		
*Comorbidity, n (%)*					
One or more	59 (76.6)	37 (75.5)	10 (71.4)	0.096 (1)	.04
Autism	4 (5.2)	3 (6.1)	1 (7.1)	0.019 (1)	.02
Intellectual disability	16 (20.8)	8 (16.3)	4 (28.6)	1.059 (1)	.13
Learning disability	16 (20.8)	11 (22.5)	4 (28.6)	0.225 (1)	.06
Language disorder	7 (9.1)	7 (14.3)	0	2.25 (1)	.19
Emotional disorder	6 (7.8)	5 (10.2)	0	1.552 (1)	.16
Disruption and impulse control disorders	16 (20.8)	7 (14.3)	3 (21.4)	0.416 (1)	.08
Epilepsy	1 (1.3)	0	1 (7.1)	–	–
Migraine	1 (1.3)	1 (2.1)	0	–	–
Developmental coordination disorders	4 (5.2)	2 (4.1)	2 (14.3)	1.907 (1)	.17
Congenital abnormalities non specified and chromosomal abnormalities	5 (6.5)	3 (6.1)	1 (7.1)	0.019 (1)	.02
Abnormal EEG	3 (3.9)	3 (6.1)	0	0.9 (1)	.12
QI ^a^	81.1 (19.9)	83.4 (20)	70.6 (20.6)	1.728 (45)	.64
*Other treatment, n (%)*					
One or more	75 (97.4)	48 (98)	13 (92.9)	0.922 (1)	.12
Counselling	1 (1.3)				
Parent training	37 (48.1)	22 (44.9)	4 (28.6)	1.198 (1)	.14
Cognitive-behavioral therapy	69 (89.6)	45 (91.8)	12 (85.7)	0.474 (1)	.09
Child training	0	–	–		
Psychodynamic psychotherapy	0	–	–		
Family therapy	0	–	–		
Other	4 (5.2)	4 (8.2)	0	1.22 (1)	.14

The sum of patients of “in treatment” and “treatment discontinuation” groups does not equal that of patients of total sample since not all patients was included in subgroup analysis. ^*a*^*:* data available for 58 patients totally and for 47 in group comparison. ^*b*^*:* Welch homogeneity correction was applied. *M* mean, *SD* standard deviation, *ASL* local Health Service office, *ADHD-C* attention deficit/hyperactivity disorder combined type, *ADHD-H* attention-deficit/hyperactivity disorder hyperactive type, *ADHD-I* attention-deficit = hyperactivity disorder inattentive type, *QI* quotient of intelligence. * *p* < 0.05

Most (92%) of patients were diagnosed with combined type of ADHD, 6.5% with the inattentive type and only one (1.3%) with the hyperactive one.

77% of patients showed one or more comorbid disorder as follows: 54.6% one, 19.5% two, 1.3% three and 1.3% four disorders. The most frequent comorbid conditions were disruption and impulse control disorders, intellectual and learning disability.

Nearly all patients (97.4%) received at least one intervention other than pharmacotherapy: 52% of patients received one other intervention, 44.2% two and 1.3% three other interventions. The most frequent interventions were cognitive-behavioral therapy and parent training.

### Bivariate associations

63 out of 77 patients had at least 12 months of follow-up after their first pharmacological administration for ADHD as of May 2022. Of these, 14 (22.2%) patients discontinued ADHD pharmacotherapy. As expected these two groups differed in the total months of ADHD pharmacotherapy (Table 1). By contrast, no significant differences were observed for age at diagnosis and age at first intake of ADHD pharmacotherapy, diagnosis, comorbidity and other treatments. Further, patients in treatment showed a higher QI mean score than patients who discontinued treatment (borderline significant *p* value of 0.091).

Considering between-groups comparisons on the CBCL, differences in externalizing and rule-breaking behaviour were borderline significant (*p* = 0.075 and *p* = 0.053, respectively) with higher symptoms in patients who discontinued vs patients who continued ADHD pharmacotherapy (Table 2). Further, patients who discontinued ADHD pharmacotherapy showed a significantly (*p* = 0.047) higher mean T total score compared to patients who continued treatment.

**Table 2:** CBCL subscales’ mean T scores of total sample and pharmacological treatment status subgroups

	Total sample ^a^ (*n* = 50) M (SD)	In treatment (*n* = 34) M (SD)	Treatment discontinuation (*n* = 6) M (SD)	*t* (df)	Cohen’s *d*
Activities	29.7 (9.9)	29.9 (11.6)	29.2 (6.9)	0.146 (38)	.07
Social	35.3 (11.5)	36.4 (13.2)	35.2 (7.7)	0.229 (38)	.10
School	34.6 (7.9)	34.4 (8.4)	37.9 (6.1)	−0.949 (38)	.42
Total competence	27.9 (13)	28.3 (15)	28.7 (8.3)	−0.054 (38)	.02
Anxious/depressed	63.5 (8.3)	63.2 (8.4)	61.3 (7.7)	0.518 (38)	.23
Withdrawn/depressed ^b^	62.2 (10.1)	63.5 (11)	61.3 (7)	0.640 (9.91)	.24
Somatic complaints	59.3 (8.6)	59 (9.1)	59 (10.8)	−0.007 (38)	.003
Social problems	65.6 (8.3)	66.7 (8.2)	61 (9.6)	1.544 (38)	.68
Thought problems	67.9 (12.6)	67.2 (13.5)	73.3 (9.3)	−1.055 (38)	.47
Attention problems	71.3 (8.3)	71.7 (9.1)	70.2 (3.7)	0.392 (38)	.17
Rule-breaking behavior	65.7 (9.3)	63.9 (9.1)	71.7 (7.1)	−2.002 (38)	.89
Aggressive behavior ^b^	71.1 (11.1)	71 (11.8)	72.7 (6.2)	−0.507 (12.61)	.17
Internalizing	63.2 (9)	63 (10.2)	65 (6.1)	−0.462 (38)	.20
Externalizing ^b^	68.5 (8.6)	67.8 (9.2)	71.3 (2.7)	−1.849 (28.87)	.52
Total score ^b^	69.1 (7)	68.4 (7.8)	71.7 (2)	−2.069 (32.87) *	.57

^*a*^ total sample of patients for which data for the CBCL was available, ^*b*^ Welch homogeneity correction was applied. * *p* < 0.05

### Logistic regression of associations with ADHD pharmacotherapy status

Considering demographic and clinical characteristics, no significant bivariate association was observed between pharmacological status and variables of interest except for a trend to a significant association with QI. Thus, model 1 was not analysed any further.

To test model 2, bivariate associations were computed considering only participants with CBCL data (*n* = 40). Patients on treatment were significantly (χ^2^ (1) = 3.971, *p* = 0.046, Cramer’s V = .32) less likely to show intellectual disability (14.7%, *n* = 5) compared to those who discontinued treatment (50%, *n* = 3) whereas no other difference was shown for demographic and clinical data. Thus, model 2 included intellectual disability and the CBCL rule-breaking behavior subscale. Results are shown in Table 3 and demonstrated significant associations between pharmacotherapy status and rule-breaking behaviour (*p* = 0.032), and intellectual disability (*p* = 0.028).

**Table 3:** Logistic regression model of factors associated with pharmacotherapy discontinuation (vs. continuation)

	Standardized estimate	OR (95% CI)	Wald statistic (df)
Intellectual disability	1.267	22.83 (1.40, 373.24) *	4.81 (1)
Rule-breaking behavior	1.600	1.19 (1.02, 1.40) *	4.58 (1)

*N* = 40. * *p* < 0.05

## Discussion

Our findings demonstrated that one in five (*n* = 14, 22% of patients with at least one-year follow-up) young patients discontinued ADHD pharmacotherapy after a mean of 12 (SD = 14.2), whereas the rest (*n* = 49, 78%) remained on treatment for a mean period of 51 months (SD = 25.3). This is consistent with the scientific literature on the topic [26, 27, 29]. Patients who discontinued pharmacotherapy were lost to follow-up owing to lack of treatment compliance, in particular, patients’ refusal to take their medication. However, information on patients’ self-reported reasons for treatment discontinuation was missing. A previous study [22] with a large Italian sample of children and adolescents with ADHD showed that 21.5% of patients discontinued treatment after two/four months of pharmacotherapy upon their parents/health professional’s decision, adverse events, loss to follow-up, lack of efficacy and other reasons.

Considering group differences according to pharmacological treatment status, a trend for group difference for IQ emerged. Patients on treatment showed a higher mean IQ score than patients who discontinued treatment. On the other hand, no significant differences were observed for age at diagnosis, age at first intake of pharmacotherapy and other demographic characteristics, diagnosis, comorbidity and other interventions. Further, considering the subsample (*N* = 40) for whom CBCL data were avaible, intellectual disability and rule-breaking behavior were associated with pharmacological treatment status. Patients with high rule-breaking behaviour and intellectual disability were more likely to discontinue ADHD treatment. These findings were unexpected since previous studies mainly showed no differences between groups [35, 40–43] or found that comorbid disorders predicted treatment continuation [26, 39]. However, Nayak et al. [41] have found evidence of a negative correlation between adherence and ADHD severity. Between-studies differences may be explained by differences in sample and methodology. Masi et al. [51] recently highlighted that intellectual disability increases the risk of adverse event, especially irritability, suggesting low tolerability to drug treatment. On the other hand, Nayak et al. [41] showed that when controlling for side effects, the association between ADHD severity and poor adherence became non-significant. It follows that intellectual disability and rule-breaking behaviour may interact with side effects and these patients experience increased irritability with drug treatment [52], or they could perceive side effects as more distressing, both leading to treatment discontinuation. Alternatively, these patients may be less compliant with treatment and/or that their family may experience relevant difficulties in drug management leading to treatment discontinuation. Furthermore, according to our clinical experience, patients with intellectual disability and their families may have high or unrealistic expectations on ADHD pharmacotherapy in improving overall cognitive functioning. They may thus be at higher risk of discontinuing treatment, at least in our sample. Finally, patients with high symptoms of rule-breaking behavior could experience pharmacotherapy as negatively affecting their personality or they could think that medications take away their personality changing the way they perceive themselves. Therefore, treatment discontinuation may also be related to the relationship between medication, behaviour and identity. However, the association between treatment status and disruption and impulse control disorders was not significant. Taken together, our findings add new knowledge to the association between psychopathology and treatment (dis-)continuation and suggest potential explanations. Studies with large samples are needed to support our findings and clarify the relationship between psychopathology and treatment (dis-)continuation using both dimensional and categorical approaches, and controlling for important covariates.

Available treatments are only partially effective in reducing ADHD symptoms [2]. Reale et al. [11] explored clinical improvements (as evaluated by clinicians using a single item of the Clinical Global Impression) after 1 year of treatment and found no significant differences between treatment group (also including a no-treatment category) in patients with ADHD only (i.e., no comorbidity) whereas a significant difference was observed between treatment groups in patients with ADHD and comorbidity showing that combined treatment (methylphenidate plus psychological intervention) and no-treatment categories where contributing the most to the chi-square value (in opposite direction) [11].

Combined treatment might thus be useful for ADHD patients with other comorbidities. A previous meta-analysis (53) of pharmacological and non-pharmacological treatment of ADHD – including 190 randomised trials that enrolled 26,114 young participants with ADHD – seems to support the superiority of behavioural therapy in combination with stimulants compared to other treatments for ADHD, both in terms of efficacy and acceptability. Our findings stress the importance of paying special attention to patients’ intelligence quotient /intellectual disability and rule-breaking behaviour to improve treatment continuation. Methodological issues, such as short follow-up, risk of bias, industry sponsorship should be carefully considered by future research (53). In point of fact, in a meta-analysis of 63 studies enrolling 11,788 young patients Riera et al. (54) found that efficacy was smaller for treatment studies in which a psychiatric comorbid disorder was an inclusion criterion, while it was larger in studies with a commercial sponsorship and showed a negative association with treatment length.

### Limitations of the study

The study was conducted with a population of participants from a geographic and cultural area, namely an ADHD reference centre located in a Southern Italian region. Thus, its results may not be generalized to other different populations or settings. The retrospective design of the study and the small sample size may also limit generalizability of results. Further, data were collected in an ADHD clinical routine management context rather than for specific research purposes. IQ was available for *n* = 58 (75.3% of the sample) and was measured using the WISC (*n* = 47. 61% of the sample; WISC-III for *n* = 3 and WISC-IV for *n* = 44), the Leiter-R (*n* = 4, 5.2% of the sample) and the WPPSI-III (*n* = 5, 6.5% of the sample). The IQ was not available for two young patients as they had been diagnosed by external neuropsychiatrists and a complete diagnostic assessment was not repeated at Scientific Institute IRCCS “*E. Medea*” (see the Statistical analysis paragraph). Use of distinct IQ scales was not associated with treatment status nor with IQ score (statistical analysis not reported in the text). Finally, the lack of pathients’s self-reported data on reasons for discontinuation, parental/family and social characteristics may limit the interpretability of our findings since adherence in young people could be determined or at least influenced by these factors [27, 41, 43].

Despite these limitations, the present study included data on patients in a clinical setting incorporating real-life variability in psychopathological symptoms at presentation (no exclusion criteria applied) supporting its ecological validity.

## Conclusions

Our findings showed that a higher frequency of intellectual disability and higher rule-breaking behaviour pre-medication in patients who eventually discontinued therapy, compared to patients who continued treatment. This highlights the need for periodical assessments monitoring difficulties with and/or reasons for poor treatment compliance as well as side effects to provide individualized care.

## Data Availability

The datasets used and/or analysed during the current study are available from the corresponding author on reasonable request.

## Abbreviations

ADHD: Attention-deficit/hyperactivity disorder
CBCL: Child behavior checklist
IQ: Intelligence quotient
